# Supplementary material for: Unraveling the Unusual Subgenomic Organization in the Neopolyploid Free-Living Flatworm Macrostomum lignano
Source: Mol Biol Evol. 2023 Nov 18;40(12):msad250. doi: 10.1093/molbev/msad250 (PMC10733133; doi:10.1093/molbev/msad250)
Supplement: msad250_Supplementary_Data [file msad250_supplementary_data.pdf]

Supplemental Material

Figure S1. IGV snapshot illustrating the construction of paralogous blocks (PBs) from chained pairwise alignments of Mlig_3_7 assembly on itself. Tracks of short-read coverage (a), chained alignments of scaffolds (b), boundaries of the PBs (c), genomic repeats (d), and gene anntotation (e) of Mlig_3_7 are shown. Color legend define the number of members within each PB.

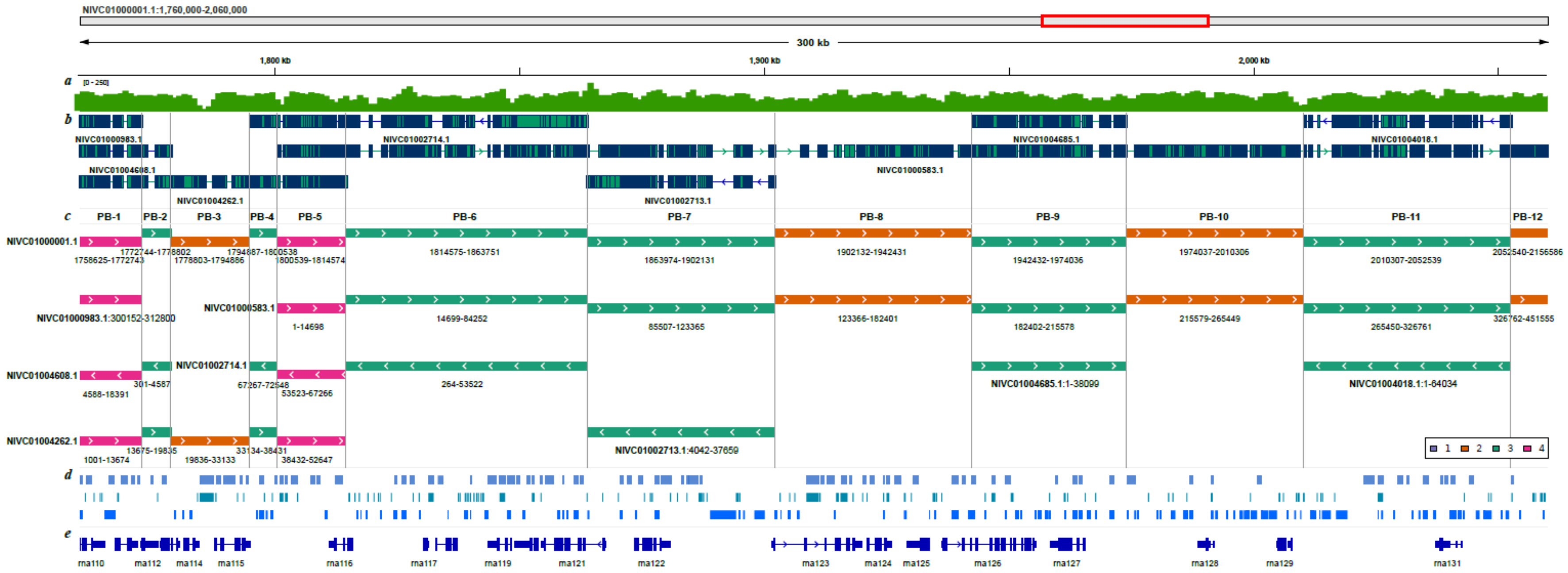

Figure S2. Karyotypes of worm-founders used for generation of worm-pools DV1_8A-E and DV1_10A-E. (A) the species-specific karyotype of *M. lignano* 2n=8. (B) the 2n=10 karyotype with additional two copies of the large chromosome MLI1. (C) the karytoype variant 2n=12 (three large and nine small metacentrcis) found in one founder of the DV1_8B worm pool. Scale bar 10 mkm.

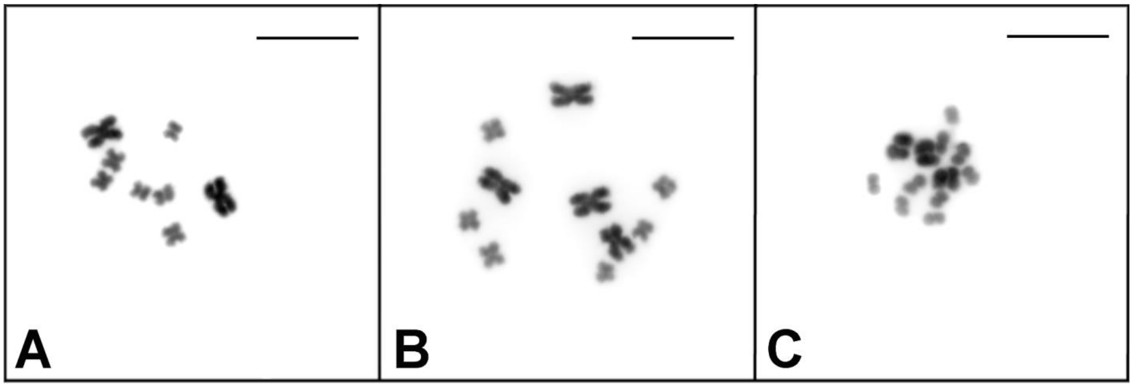

Figure S3. Dependence of bivariate k-mer frequency distribution on GC-content of k-mers. The four density plots of 35-mer frequencies in DV1_8 and DV1_10 datasets are similar to the one provided in Figure 3, except that they were built for subsets of 35-mers containing a given range of GC bases: 3-11, 12-17, 18-23, 24-35.

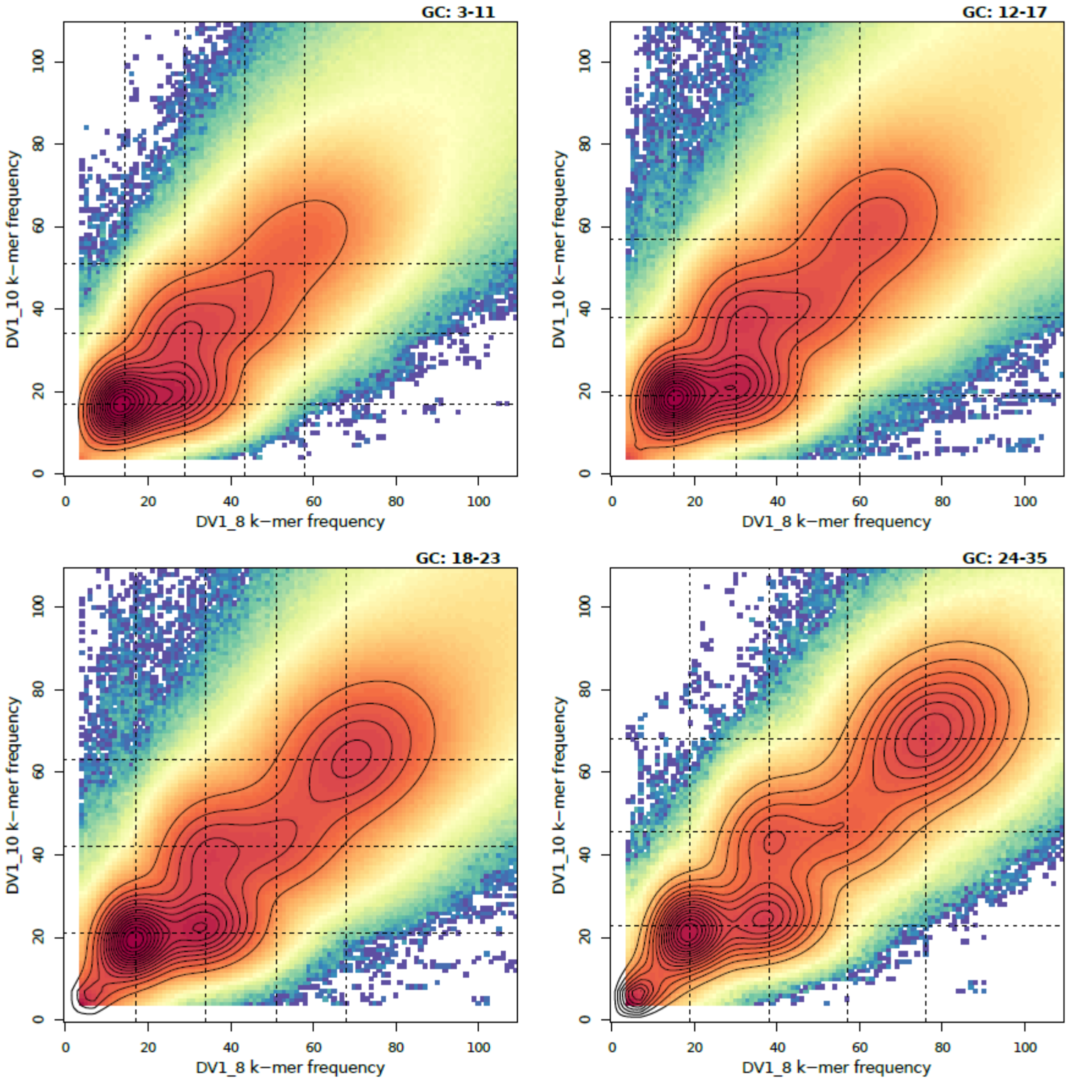

Figure S4. Bimodal distribution of the $\log_2$-ratio of read coverage (DV1_8 to DV1_10, $\log_2(R_{RC})$).

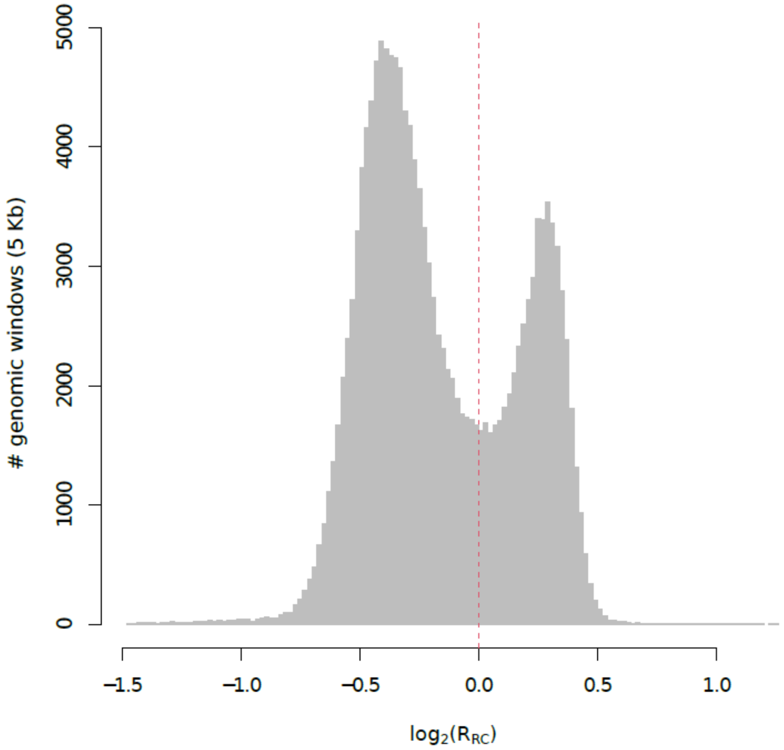

Figure S5. Consistency of genome-wide coverage profiles among the sequenced DV1_8 and DV1_10 genomic libraries. For each replicate, reads were mapped on the reference genome, and the mean depth of coverage was computed for all 5-kb genomic windows. Scatterplots show the resulting profiles compared to the averaged profile of all replicates. The color scale denotes the density of genomic windows.

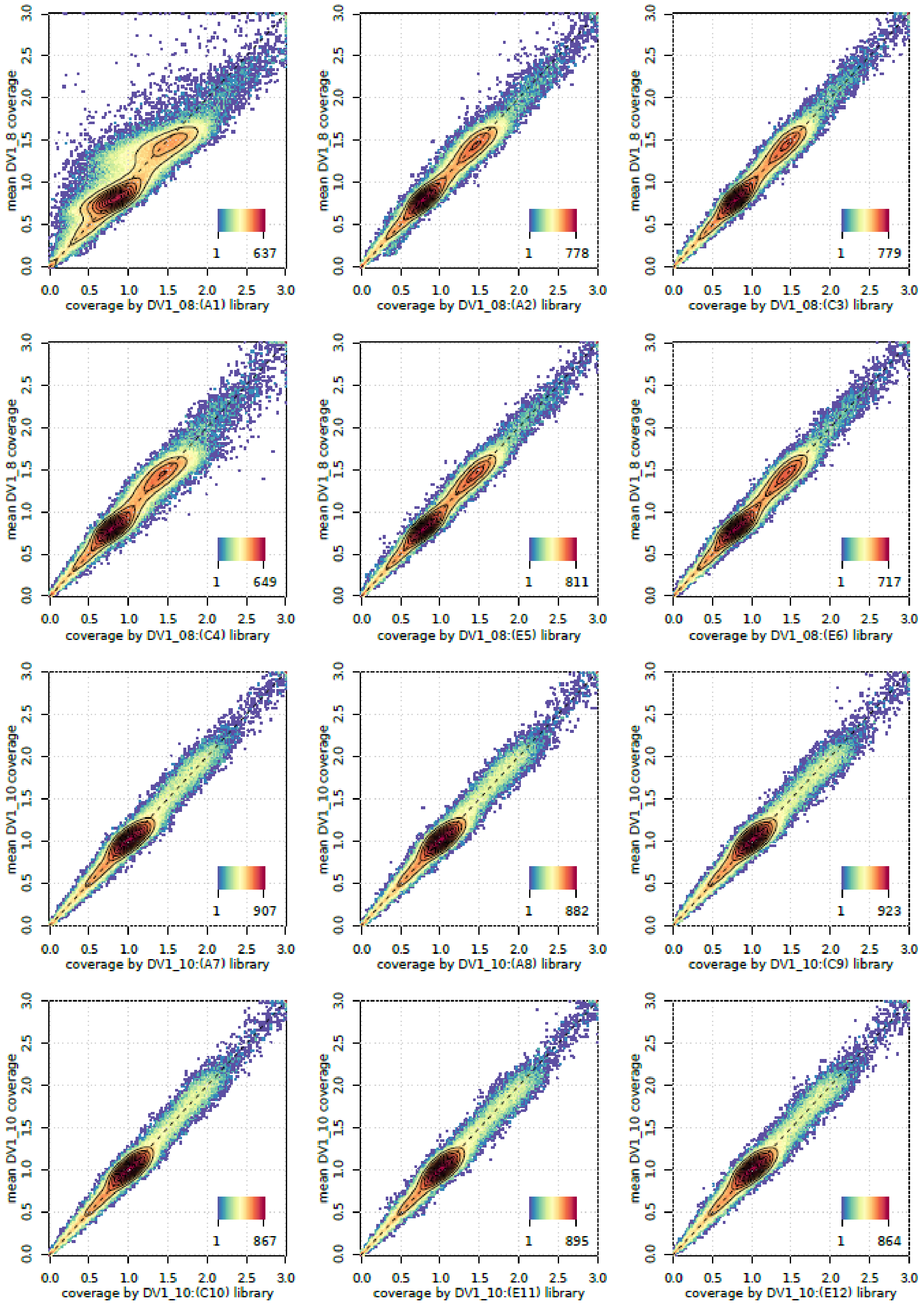

Figure S6. Multi-mapping bias associated with incogruence between subgenome contents of the reference assembly and the sequenced DV1_10 genome. The left 2D-density plot shows DV1_8 versus DV1_10 coverage profiles of the entire genome assembly (the same as in Figure 2A). The right 2D-density plot shows the same distribution but for the unique fraction of genome selected using mappability of 1 according to the genmap algorithm. In the case of the entire assembly, the ratio between the peaks in DV1_10 is 1.81 instead of the theoretical 2-fold difference, however removal of multi-mappable genomic loci results in nearly theoretical ratio of 1.97.

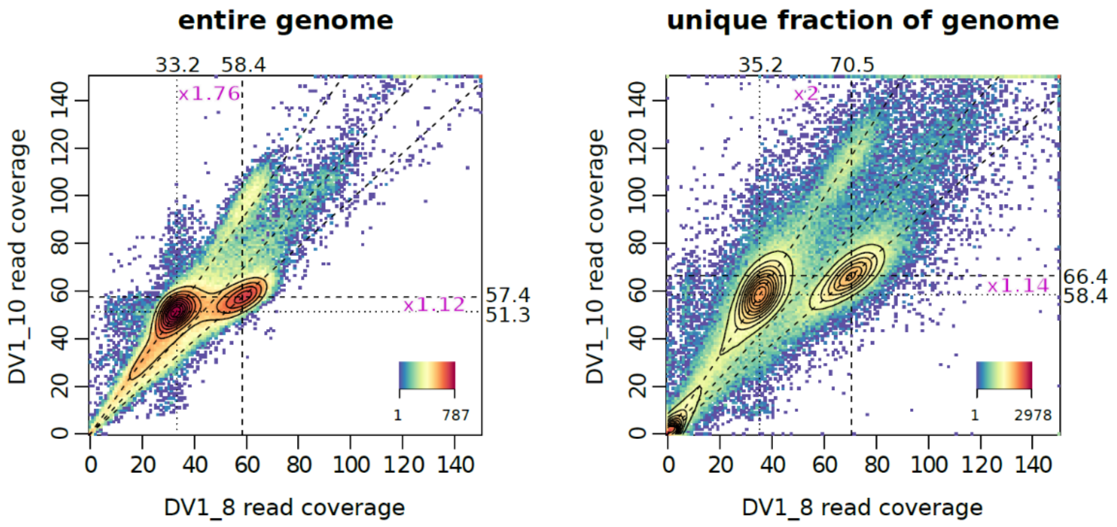

Figure S7. The abundance of predicted classes and families of DNA repeats in the Mlig_3_7 assembly classified into L and S subgenomes. The barplots illustrate the proportion of repetitive DNA in two subgenomes: repeats with assigned classes (left) and top-50 most frequent families of repeats from "Unknown" class (right). The numbers to the right of the bars are the ratios of corresponding repeats in two subgenomes.

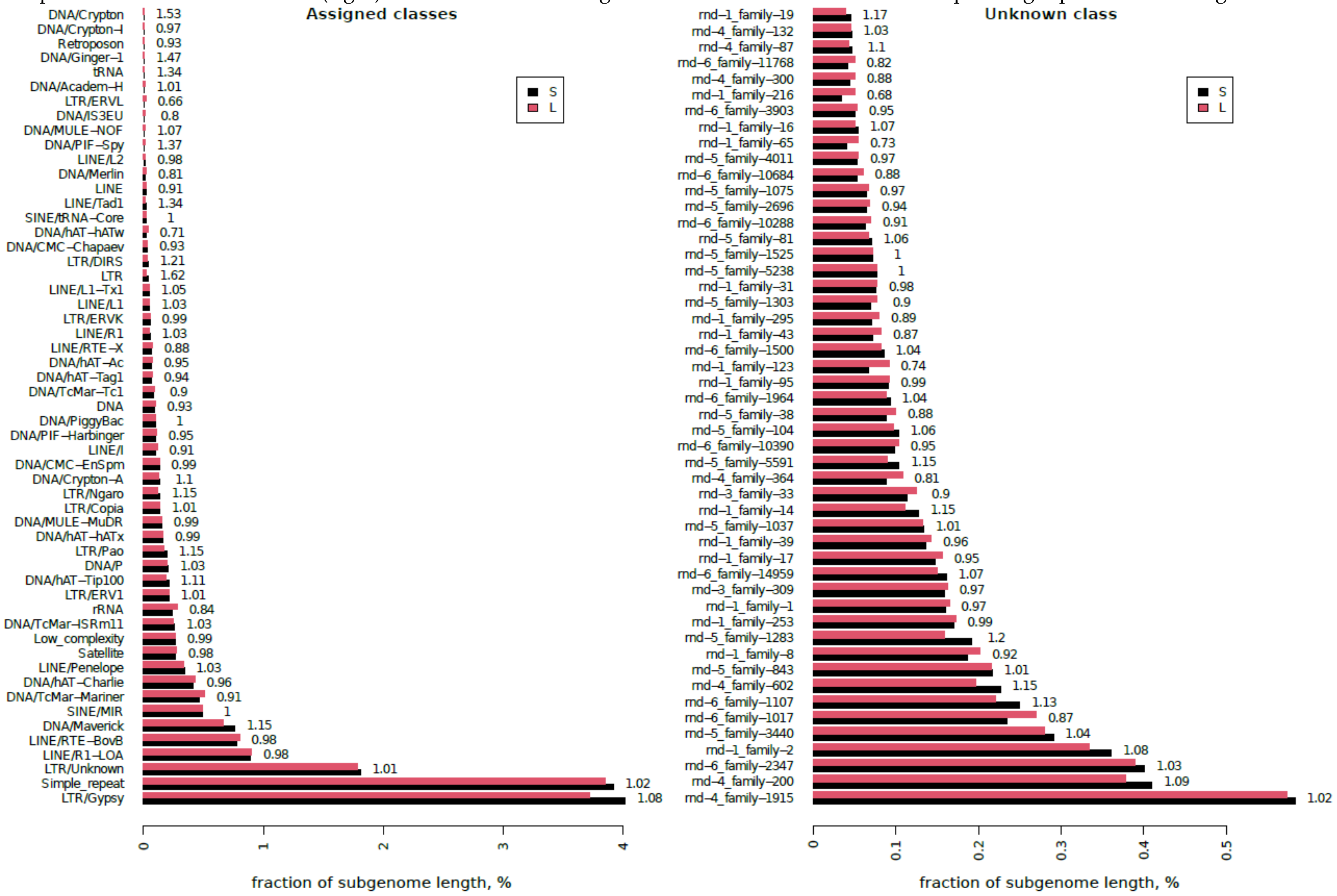

Table S1. Heterozygosity parameters used in GenomeScope2.0 «hexaploid» model and their compatible transition into «tetraploid» model. Additional parameters ε_1 and ε_2 were incorporated to resolve ambiguous transition from «aaaabb» and «aaabbb» forms into «aaab» and «aabb». The forms «aabcde» and «abcdef» cannot be represented in «tetraploid» state.

Heterozygosity forms		Allele ratio		Parameters		Equations between two sets of parameters	Estimated rates of heterozygosity	
hexa	tetra	hexa	tetra	hexa	tetra		hexa	tetra
aaaaaa	aaaa	(6)	(4)	-	-	-	-	-
aaaaab	aaab	(5:1)	(3:1)	r_1	q_1<br>q_2	$q_1=r_1+\varepsilon_1 r_2+\varepsilon_2 r_3$<br>$q_2=(1-\varepsilon_1)r_2+(1-\varepsilon_2)r_3$	0.00 %	1.03%<br>1.22%
aaaabb	aaab<br>aabb	(4:2)	(3:1)<br>(2:2)	r_2			2.15 %	
aaabbb	aaab<br>aabb	(3:3)		r_3			0.10 %	
aaaabc	aabc	(4:1:1)	(2:1:1)	r_4	q_3	$q_3=r_4+r_5+r_6$	0.01 %	0.36%
aaabbc	aabc	(3:2:1)		r_5			0.00 %	
aabbcc	aabc	(2:2:2)		r_6			0.35 %	
aaabcd	abcd	(3:1:1:1)	(1:1:1:1)	r_7	q_4	$q_4=r_7+r_8$	0.00 %	0.00 %
aabbcd	abcd	(2:2:1:1)		r_8			0.00 %	
aabcde	-	(2:1:1:1:1)	-	-	-	-	-	-
abcdef	-	(1:1:1:1:1:1)	-	-	-	-	-	-

Table S2. Interpretation of copy-number peaks from 3D spectra of k-mer frequencies in terms of genome organization in DV1_8 and DV1_10 sublines. Headers contain (*i*) proposed genome composition in terms of subgenomes, (*ii*) possible copy-number values satisfying this composition, and (*iii*) the ratios of allelic variants that contribute to the corresponding copy number. The cells contain symbols of subgenomes that contribute to the corresponding copy-number peaks of both DV1_8 and DV1_10 genomes. Equal sign means identity of subgenomes in a given position; unobservable states are marked by a dash.

**DV1_10:** $SSL_1L_1L_2L_2$	×6	(**6**)	-	-	-	[$S=L_1=L_2$]
	×4	(**4**:2)	-	[$L_1=L_2$]	[$S=L_1$]<br>or<br>[$S=L_2$]	-
	×2	(**2**:**2**:**2**)<br>(4:**2**)	[L_1]<br>or<br>[L_2]	[S]	-	-
i	*ii*	*iii*	(2:**1**:**1**)<br>(3:**1**)	(**2**:1:1)<br>(**2**:**2**)	(**3**:1)	(**4**)
		ii	×1	×2	×3	×4
		i	**DV1_8:** SSL_1L_2			

Table S3. Generation of worm pools DV1_8A-E and DV1_10A-E.

Subline	ID pool	Parental worms-founders	Karyotype
DV1_8	8A	20 worms	2n=8 (100%)
	8B	20 worms	2n=8 (95%)<br>2n=12 (5%)
	8C	20 worms	2n=8 (100%)
	8D	20 worms	2n=8 (100%)
	8E	20 worms	2n=8 (100%)
DV1_10	10A	20 worms	2n=10 (100%)
	10B	20 worms	2n=10 (100%)
	10C	20 worms	2n=10 (100%)
	10D	20 worms	2n=10 (100%)
	10E	20 worms	2n=10 (100%)
